# Antiseptic Science in Chicago

**Published:** 1904-07

**Authors:** 


### Editorial.

### ANTISEPTIC SCIENCE IN CHICAGO.

IN January last a paper was read before the Chicago Dental Society, by Hermann Prinz, M.D., D.D.S., of St. Louis, on "A Few Facts concerning the Action of Antiseptics." This paper was given to readers of dental subjects in the *Dental Review,* of Chicago, May 15, 1904. The essay was an able *résumé* of the subject, largely made up, outside of the author's own opinions, with the history of the subject and the theories and investigations of other minds. Very little original work by the writer appears in the article. This, however, did not militate against its interest, but rather enhanced its value as a contribution to the knowledge of the subject. It brought forth an interesting discussion in which the author of the paper naturally took an active part.

In the course of his remarks he went out of his way to scarify the present writer, because in a recent review he had the audacity to express certain convictions regarding the action of silver nitrate on tissue.

The remarks of Dr. Prinz, as reported, were as follows: "As to metal salts, I have looked into them very thoroughly. It is almost incredible to believe that a man like Dr. Truman, of Philadelphia, stands by a statement to-day that he made six years ago. In one of the latest numbers of the INTERNATIONAL DENTAL JOURNAL, in reviewing a book on modern therapeutics by Stevens, he attacks the statement made by Stevens as to the limitation of silver nitrate in tissue, saying that it does not exist; that he has demonstrated this in test-tubes, etc. I have worked in the mouths of poor patients; I have taken the lower second molar, in which there was deep caries; placed in there pure nitrate of silver, sealed it in for months, and after a while extracted the tooth, and it has shown distinctly the limitation of nitrate of silver. My observations have convinced me that nitrate of silver is self-limiting in its action. It precipitates albumin and silver albumin will not allow the smallest particle of silver to penetrate any farther. It will not do so, no matter what the test-tube shows. Living subjects are the ones in which to make these experiments, and experiments show that there is a resisting force in the tissue. There it stops. There is no force behind it."

The personal part of this quotation is not regarded as of any importance by this writer. The editor of this journal has long since ceased to reply to mere personalities, and would have allowed this to sink into forgetfulness were it not for the fact that this paragraph contains either important truths, or serious errors, of grave concern to all practising dentists. Is the dental profession prepared to accept the dictum that silver nitrate can be applied to a tooth *ad libitum,* and that without producing discoloration? If they are willing to accept Dr. Prinz's dogmatic assertion as the truth, then they can apply this metal salt without fear to any and all teeth with which we have to deal; but if they doubt his conclusions, they will agree with the writer and wait further investigations before risking their reputations on the mere statement that silver nitrate is self-limiting.

Dr. Prinz has poor patients. That is commendable. He takes a "lower second molar and applies silver nitrate and seals it there for months." He speaks of "deep caries," but does not state whether the pulp was exposed, gangrenous, or whether he applied the silver salt to a clean, dry canal, or whether the application was in crystal form or solution. It is taken for granted that the pulp was not exposed, and that he placed the silver nitrate in the cavity in crystals upon the open ends of the tubuli of the dentine. After months he extracted the tooth. It was certainly kind in this poor patient to submit to this mutilation in the cause of science. It is another item for the Antivivisection Society to record, and might be of value to the Society for Prevention of Cruelty to Children, providing St. Louis supports such an organization. This question can best be settled by those most interested. The writer would suggest to Dr. Prinz that when he makes further experiments in this direction, he take a number of vital teeth, and not confine his work to one. Place the silver nitrate in the cavity in solution and not in crystals. Saturate cotton with it so that it will remain for a definite period in contact with the living protoplasm of the tubes. If after several months he finds, upon extraction and sectionizing these teeth, that the silver salt has not coagulated the organic material, he will be able to assert, with some degree of positiveness, that silver nitrate "precipitates albumin and silver albumin will not allow the smallest particle of silver to penetrate any farther." Silver nitrate is not deliquescent. It will remain as he placed it, unchanged indefinitely, and it is doubtful whether the salt thus placed will penetrate deeply. It has also to be proved to what extent vitality resists the progress of a coagulant. It has been fully and completely demonstrated that dead protoplasm offers no obstruction to its progress in the tabulated structure of dentine. This the writer demonstrated years ago, and Dr. Prinz can read the results as published at the time, if not already familiar with them.

The evidence furnished by Dr. Prinz, in his one tooth, that vitality is a resisting force is not sufficient. The evidence offered by Dr. Stebbins is probably the only test of much value in this direction, and this is not conclusive, for while working on poor children he failed to extract the teeth. Dr. Stebbins, then unknown to fame, called the writer's attention to a group of children with him, while the latter was in attendance at the American Dental Association at Saratoga. His statement that he had been using silver nitrate in some of the cavities for a period of six years was received by the writer with marked evidence of incredulity. The facts, however, were too positive to be set aside without further and more careful examination. Some of these deciduous teeth were vital, and, as far as the observer could determine from external examination, they were not seriously discolored. This was, then and now, attributed to the fact that Dr. Stebbins had *carefully removed all excess* of the silver nitrate, to avoid, if possible, any deep penetration and, at the same time, secure the antiseptic property of the agent. In this he succeeded, and in teeth exposed to the destructive action of the fluids of the mouth there was no evidence of deterioration from caries. Dr. Stebbins had, however, no thought of determining the resisting power of living tissues. That this is a factor to be considered requires no argument, but the extent of the resisting force has, as yet, not been formulated, and the writer may be pardoned if he is sceptical as to Dr. Prinz's success in proving it to be an assured fact. It seems to the writer that the essayist's descent from the sublimity of scientific statements, in his paper, to the paragraph quoted from the discussion, is a remarkably good illustration of a descent from the elevated in writing to the bathos of speech.

Some years since, when the question of the penetration of coagulants was a warm subject for dispute in our national conventions, the action of silver nitrate came up for consideration in the American Dental Association. Those who advocated its use in pulp-canals took practically the same position as Dr. Prinz,—that it would not penetrate the tubules, that its penetration would be prevented by its own coagulant, etc. Specimens were handed around to prove this; human teeth in microscopic sections. The result had its amusing side for those who had contended that silver nitrate was not self-limiting, for all of these sections clearly demonstrated that it had entered the tubes to a considerable extent; in fact, was only self-limiting up to the quantity used. So far as it went, it effectually discolored the protoplasm in the tubes.

The writer is aware that mere opinions are not worthy of much consideration upon a matter of this character, but there must have been some clinical evidence that led to the universal teaching, for a half-century and longer, that silver nitrate would discolor tooth-substance if brought in contact with organic matter. This was the universal opinion; it was universally taught and as universally accepted. When one as prominent in professional circles as the writer of the paper mentioned takes great pains to assure the rising professional generation that it will not discolor, that it will not penetrate beneath the surface, that it can be used with safety, and that "silver is one metal of all others, no matter what statements are made to the contrary, which is best tolerated by the tissues," he is bound to prove it beyond all fear of successful criticism.

The writer's experiments, alluded to by the essayist, were not made especially to prove the penetrating power of silver nitrate, but they were conducted through months and in a variety of ways to demonstrate the action of coagulants in the presence of albumin. Silver nitrate was one of many. In regard to this the writer entertained the opinions universally held by writers, including the author of the paper, that the action of this metal salt was superficial. To his great surprise he found that in sealed capillary tubes filled with egg albumin the penetration was continuous until the entire mass was coagulated. The experiments were then transferred to human teeth. The same results followed, many sections showing deep discoloration throughout the tubulated structure. If these experiments are not satisfactory to Dr. Prinz, it belongs to him to originate more positive methods. It seems to the writer that the older theories that have attempted to describe its action in the presence of albumin need revision, and it might be instructive to the coming generation of dentists if Dr. Prinz would extend his laboratory experiments from one tooth to many.

This sort of teaching has given dentists the courage to use silver nitrate indiscriminately; indeed, we find one prominent writer recommending it as an occasional mouth-wash in solution. A conservative estimate of its value is, therefore, greatly needed.

The writer still remains of the opinion that silver nitrate is *not self-limiting,* and that the reason why it does not always penetrate deeply is simply that the agent was used in too limited a quantity to permit active coagulation upon the deeper layers of albumin. The question is, therefore, one of quantity in solution and continuance of action.

